# High Prevalence of Psychological Comorbidities and Functional Neurological Symptoms in Women With Urinary Retention

**DOI:** 10.1097/JU.0000000000003983

**Published:** 2024-05-10

**Authors:** Caroline Selai, Cheng-hung Lee, Sara Simeoni, Mahreen Pakzad, Eileen Joyce, Pany Petrochilos, Khadija Rehrou Rantell, Vasile Boico, Jalesh N. Panicker

**Affiliations:** 1 Department of Clinical and Movement Neuroscience, UCL Queen Square Institute of Neurology, Faculty of Brain Sciences, University College London, London, United Kingdom; 2 Department of Uro-Neurology, The National Hospital for Neurology and Neurosurgery, Queen Square, UCLH NHS Foundation Trust, London, United Kingdom; 3 Department of Neuropsychiatry, The National Hospital for Neurology and Neurosurgery, Queen Square, UCLH NHS Foundation Trust, London, United Kingdom; 4 Education Team, UCL Queen Square Institute of Neurology, London, United Kingdom; 5 Department of Brain Repair and Rehabilitation, UCL Queen Square Institute of Neurology, Faculty of Brain Sciences, University College London, London, United Kingdom

**Keywords:** Fowler’s syndrome, chronic idiopathic urinary retention

## Abstract

**Purpose:**

Chronic idiopathic urinary retention (CIUR) in young women is poorly understood and a probable etiology is established only in around 40%, most commonly a primary disorder of external urethral sphincter relaxation, sometimes referred to as Fowler’s syndrome. A high prevalence of psychological and functional comorbidities is reported, however these have been poorly characterized.

**Materials and Methods:**

Women consecutively referred for the assessment and management of CIUR were evaluated cross-sectionally for 13 psychological/behavioral domains using a structured clinical interview: depression, anxiety, post-traumatic stress disorder (PTSD), other psychiatric history, functional neurological disorder, other functional syndromes, childhood and adult trauma, personality disorder, and self-harm (ever/current).

**Results:**

A total of 91 women (mean age [SD]: 34 [11] years) were evaluated. Women with Fowler’s syndrome (n = 69) were younger (mean age [SD]: 32 [9] vs 40 [13] years) than women without Fowler’s syndrome and reported shorter mean duration of urinary symptoms (mean [SD]: 5 [6] vs 10 [9]). A high prevalence of psychiatric and psychological comorbidities was reported (97%) including current depression (77%), current anxiety (78%), and PTSD (32%). A high prevalence of functional neurological disorder (56%) and other functional symptoms (65%) was also reported. Self-harm was reported in (14%) and personality disorder in 16%. Childhood trauma was reported in 35% of women.

**Conclusions:**

Young women with CIUR report a high burden of psychiatric disorders, affective symptoms, trauma, PTSD, self-harm, and functional neurological disorder, particularly in those with Fowler’s syndrome. These factors can undermine the engagement with health care professionals and affect management and should therefore be addressed during the urological assessment.

Chronic idiopathic urinary retention (CIUR) in young women is poorly understood and prospective studies suggest that despite extensive urological and neurological investigations, a probable etiology may be established only in around 40% of women, most commonly a primary disorder of external urethral sphincter relaxation, sometimes referred to as Fowler’s syndrome.^1^ The condition is characterized by a poorly relaxing urethral sphincter that manifests as functional bladder outlet obstruction, associated with an abnormally elevated urethral pressure profile and/or abnormal urethral sphincter electromyography (EMG).^2^ Treatment options for women with chronic urinary retention are limited, however women with Fowler’s syndrome show a favorable response to sacral neuromodulation^3^ and, more recently, urethral sphincter injections of botulinum toxin.^4^

In recent years, studies have suggested the co-occurrence of medical, psychiatric disorders, psychological comorbidities, and functional disorders in women with CIUR. The most common psychological comorbidities reported were affective disorders such as depression and anxiety.^5-8^ Furthermore, there is a growing recognition of functional disorders, whose origin arises primarily from a disorder of nervous system functioning rather than a clearly identifiable pathophysiological disease, and their dynamic interrelationship with psychological comorbidities.^9^ Functional neurological disorder is characterized by clinical features that indicate that symptoms arise from a functional rather than structural disorder.^10,11^ Previously these symptoms have been described using a range of terms including “hysterical,” “conversion,” and “somatization.” Examples include functional movement disorders, persistent perceptual postural dizziness, functional cognitive disorder, and functional seizures.

The extent of psychological comorbidities and functional neurological symptoms in women with CIUR, and their impact, remains unclear and the aim of this study was to assess their prevalence and to explore possible relationships between these comorbidities and lower urinary tract (LUT) dysfunction.

## METHODS

In this cross-sectional study, consecutive women referred to a tertiary center teaching hospital for assessment and management of chronic urinary retention, that is, the complaint of chronic or repeated inability to empty the bladder, despite the ability to pass some urine,^12^ underwent a standardized multidisciplinary assessment.^1^ Neurological and urological causes were evaluated and women underwent noninvasive uroflowmetry and measurement of post-void residual volumes, and invasive multichannel urodynamic studies, including filling cystometry (medium fill at 50 mL/min) and pressure-flow studies (Medical Measurement Systems, Dover, New Hampshire) following International Continence Society Good Urodynamic Practices.^13^ The urethral pressure profilometry (UPP) was performed (n = 62) as per the Brown-Wickham method using a fluid-filled catheter, with continuous perfusion at a rate of 5 mL/min using an Alaris GS syringe driver (stepper-motor, occlusion pressure limit set to 200 cm H_2_0). Concentric needle EMG of the striated urethral sphincter was performed (n = 79; Sierra Summit EMG/EP, Medevolve) and interpreted independently by a uro-neurologist (J.N.P., S.S.). The EMG recording was analyzed qualitatively for the presence of decelerating bursts and complex repetitive discharges heard over the audio-amplifier of the EMG machine. EMGs were defined as abnormal in the presence of decelerating bursts.^14,15^ Patients with either an abnormally elevated UPP or distinctly abnormal urethral sphincter EMG were diagnosed to have Fowler’s syndrome.^1^

Patients were referred to the newly established psychology service (within the uro-neurology team) by members of the team. Women underwent a semistructured clinical interview by a chartered psychologist (C.S.).^16-18^ The first part of the interview included an evaluation of 12 domains of physical and psychological well-being (Supplementary Appendix 1, https://www.jurology.com). The questions about current anxiety and depression were based on the 2-item GAD-2 which screens for generalized anxiety disorder^19^ and the 2-item PHQ-2 screen for depression.^20^ The second part of the interview explored current psychological comorbidities, psychoeducation, and sources of support.

This was an in-depth clinical interview with questions on demographics, physical health conditions, description and duration of somatic symptoms (in years), social and family situation, current psychological well-being, past medical and family history, school, and work/career. After a general question: did you experience any challenges/stressful experiences, sensitive exploration of trauma was informed by the literature.^21^ The headings in the Supplementary Appendix (https://www.jurology.com) are a list of the areas that the interview covered. Respondents were not pressed to give details of the childhood or adult trauma, although some spontaneously disclosed childhood sexual abuse, physical abuse and/or neglect, and, in adulthood, domestic violence, sexual assault.

Respondents were asked if they had either been given a diagnosis of functional neurological disorder or had what had been described as functional neurological symptoms and/or other functional disorders (with chronic fatigue syndrome [CFS], fibromyalgia, and irritable bowel syndrome [IBS] given as examples of the latter).

The Queen Square Clinical Audit Committee approved this study as a service evaluation (registration No. 67-202122-CA) and assessments were conducted as part of a standard care pathway in the department.^1^

Continuous data were summarized descriptively using mean and standard deviation (SD) or median and range, depending on the data distribution. Categorical data were summarized using count and percentages. Comparisons between women with Fowler’s syndrome and those without Fowler’s syndrome were performed using the chi-square test or Fisher’s exact test where appropriate, or independent *t* test depending on the scale of the outcome variables (numerical or categorical). *P* values and corresponding 95% confidence intervals (CIs) were derived using either a 2-sample independent *t* test or test for the equality of proportions using large-sample statistics.

## RESULTS

In total 186 consecutive females attended clinic for the management of chronic urinary retention between January 2018 and September 2019, and 91 (mean age [SD] 34 [11] years) consented and were included in this study. Mean (SD) duration of LUT symptoms was 7 (7) years. Table 1 provides a summary of comparisons of presenting urinary symptoms and urodynamic findings by Fowler’s syndrome status. A total of 69 (76%) patients received a diagnosis of Fowler’s syndrome based on urethral sphincter EMG and/or UPP findings. The cohort with Fowler’s syndrome was younger and had a shorter duration of LUT symptoms. No neurological or urological cause for urinary retention could be established in the remaining patients. Overall, 59 (64%) of patients were in complete retention, with a higher proportion in the Fowler’s syndrome cohort compared to the cohort without Fowler’s syndrome (69% vs 50%).

**Table 1.** Summary of Comparison of Lower Urinary Tract Symptoms and Investigation Findings in 91 Females Presenting With Chronic Idiopathic Urinary Retention by Fowler’s Syndrome Status

	Fowler’s syndrome		Overall	Difference (95% CI)	*P* value^a^
	Yes N = 69	No N = 22	N = 91		
Age, mean (SD), y	32 (9)	40 (13)	34 (11)	−8 (−13, −3)	**.001**
LUT symptoms					
Duration of LUT symptoms, mean (SD), y	5 (6)	10 (9)	7 (7)	−5 (−8, −1)	**.008**
Urinary urgency, No. (%)	18 (26)	5 (23)	23 (25)	3% (−17%, 24%)	.752
Urinary frequency, No. (%)	17 (25)	4 (18)	21 (23)	6% (−13%, 26%)	.531
Incontinence, No. (%)^b^	19 (28)	7 (32)	26 (29)	−4% (−26%, 18%)	.699
Incomplete urinary retention, No. (%)	48 (69)	11 (50)	59 (64)	19% (−5%, 42%)	.113
Type of catheterization, No. (%)					
CISC	39 (56)	12 (57)	51 (56)	−1% (−26%, 23%)	.908
Indwelling catheter (urethral [n = 7] or suprapubic [n = 11])	12 (17)	6 (29)	18 (20)	−11.43% (−33%, 10%)	.249
Investigations					
Urodynamics findings (n = 71), No. (%)					
Acontractile detrusor	35 (64)	9 (56)	44 (62)	7% (−20%, 35%)	.592
Bladder outlet obstruction	13 (24)	4 (25)	17 (24)	−1% (−25%, 23%)	.910
Other finding^c^	7 (12.3)	3 (19)	10 (14)	−6% (−27%, 15%)	.542
UPP (n = 62), No. (%)					
Abnormal^d^	49 (86)	1 (20)	50 (80.7)	66% (30%, 102%)	**< .001**
Urethral pressure profile (n = 62), mean (SD), cm H_2_O	90 (31)	45 (23)	86 (33)	45 (16, 73)	**.003**
Urethral sphincter EMG (n = 79), No. (%)					
Abnormal	53 (80)	0	53 (67)	80% (71%, 90%)	**< .001**

Abbreviations: CISC, clean intermittent self-catheterization; EMG, electromyography; LUT, lower urinary tract; SD, standard deviation; UPP, urethral pressure profile.

a *P* values and confidence intervals derived using independent *t* test or test for proportions using large sample statistics.

b Urgency urinary incontinence, stress urinary incontinence, and overflow urinary incontinence.

c Normal (n = 4), inhibited void (n = 3), bladder outlet obstruction and acontractile detrusor (n = 3).

d Value elevated greater than expected for age (1).

From the investigations, we excluded a mechanical cause for obstruction. The findings of UPP or EMG suggested the obstruction was functional from neuromuscular causes and we concluded a primary disorder of urethral sphincter relaxation (Fowler’s syndrome). Unfortunately, widely accepted validated urodynamic criteria for bladder outlet obstruction in women are lacking and hence the diagnosis was made based on the findings in fluoroscopy (lack of opacification in the distal urethra or involuntary contractions of the sphincter) or the finding of a high pressure, low flow in the pressure flow study during urodynamics. The diagnosis from the urethral pressure profile was used to corroborate this finding when the diagnosis was unclear. The uroflowmetry and post-void residual data are presented in a separate Supplementary Table (https://www.jurology.com).

The comparisons of the psychological assessment by Fowler’s syndrome status are presented in Table 2. Overall, 97% (n = 88) of patients with CIUR were found to have comorbidities. The most common psychiatric comorbidities were anxiety (around 78% to 89%) and depression (around 77% to 87%). We have looked at the proportion of patients with depression in the subgroup of patients with complete retention. For the subgroup of patients with complete retention (n = 32), the proportion of patients with depression (ever) in those with Fowler’s syndrome was double that of those without Fowler’s syndrome (67% vs 33%).

**Table 2.** Summary of Comparisons of Comorbidities in 91 Females Presenting With Chronic Idiopathic Urinary Retention by Fowler’s Syndrome Status

	Fowler’s syndrome		Overall	Difference (95% CI)	*P* value^a^
	Yes N = 69	No N = 22	N = 91		
Any comorbidities, No. (%)	66 (96)	22 (100.00)	88 (97)	−4% (−9%, 0.5%)	.320
Psychiatric comorbidities					
Psychiatric history, No. (%)	50 (72)	21 (95)	71 (78)	−23% (−37%, −9%)	**.023**
Depression ever, No. (%)	58 (84)	21 (95)	79 (87)	−11% (−24%, 1%)	.169
Depression current, No. (%)	53 (77)	17 (77)	70 (77)	−0.5% (−21%, 20%)	.964
Anxiety ever, No. (%)	60 (87)	21 (95)	81 (89)	−8% (−20%, 3%)	.267
Anxiety current, No. (%)	53 (77)	18 (82)	71 (78)	−5.00% (−24%, 14%)	.621
Post-traumatic stress disorder, No. (%)	20 (29)	9 (41)	29 (32)	−12% (−35%, 11%)	.296
Functional symptoms					
Functional neurological symptoms, No. (%)	43 (62)	8 (36)	51 (56)	26% (3%, 49%)	**.033**
Functional syndromes, No. (%)	47 (68)	12 (55)	59 (65)	14% (−10%, 37%)	.246
Other comorbidities					
Self-harm ever, No. (%)	25 (36)	9 (41)	34 (37)	−5% (−28%, 19%)	.693
Self-harm recent, No. (%)	11 (16)	2 (9)	13 (14)	7% (−8%, 22%)	.424
Personality disorder, No. (%)	14 (20)	1 (5)	15 (16)	16% (3%, 29%)	.083
Childhood trauma, No. (%)^b^	24 (35)	8 (36)	32 (35)	−2% (−25%, 21%)	.892
Adult trauma, No. (%)	28 (41)	13 (59)	41 (45)	−19% (−42%, 5%)	.129

a *P* values and confidence intervals derived using independent *t* test or test for proportions using large sample statistics.

b Sexual abuse reported in 10 patients, 6 with Fowler’s syndrome.

Other common comorbidities included adult trauma (45%) and self-harm (37%). Just over a third reported a history of childhood trauma (35%).

Though overall psychiatric illness prevalence was greater in patients without Fowler’s syndrome, there were no significant differences in specific comorbidities between the 2 groups.

## DISCUSSION

In this study, consecutive women referred for the assessment and management of chronic urinary retention underwent an assessment of psychological comorbidities using a structured assessment. Only half of the patients with chronic urinary retention (91 out of 186) could be consented to take part in this study. When we first set up the psychology service, patients were invited to speak to the psychologist directly after their appointment. However (1) many patients had travelled some distance to the clinic in London and were not able to stay on, (2) some patients did not think that speaking to a psychologist was relevant to their voiding difficulties, and (3) many patients with functional neurological symptoms reported previous negative experiences, such as being disbelieved.

The very high percentages of respondents in this study who reported depression and/or anxiety may indicate bias in this study; that is, particularly complex patients are referred to this center for tertiary referral. Second, the data were based on a semistructured clinical interview, and only brief 2-item screening measures for anxiety and depression were used in this study.

Previous studies have demonstrated a high prevalence of affective symptoms in patients with LUT symptoms. Amongst patients with a clinical diagnosis of overactive bladder (OAB), severe or moderate depression has been reported in up to 60% of patients, whereas moderate/severe anxiety in 62%.^22^ In women with urge incontinence, the prevalence of anxiety and depression was reported to be 57% and 38%, respectively.^23^ A systematic review demonstrated an association between depression and OAB symptoms, and between anxiety and OAB.^24^ The prevalence of depression in patients attending general practice in the United Kingdom is up to 10%, rising to 20% amongst patients with chronic medical conditions.^25^ The prevalence of anxiety in a general population has been estimated to be only 3.6%.^26^ The results of this study suggest that the prevalence of these affective symptoms are significantly greater in women with CIUR. In a multicenter randomized clinical trial evaluating sacral nerve stimulation for refractory voiding dysfunction, 73% of patients were reported to have depression.^27^

A retrospective review of medical notes of women with Fowler’s syndrome suggested that 30% of women were reporting symptoms of anxiety, depression, or obsessive compulsive symptoms.^5^ In our study, using the 2-item GAD-2 which screens for generalized anxiety disorder^19^ and the 2-item PHQ-2 screen for depression,^20^ 77% of women reported experiencing depression and 78% of women were found to have anxiety. Particularly concerning was the high prevalence of patients who had ever self-harmed (37%) and had recently self-harmed (14%; ie, within the previous 6 months). These figures are far greater than in the general population where the prevalence of self-reported lifetime nonsuicidal self-harm has been reported to be 6.4%.^28^

In this study, 15 women (16%) reported they had previously been given a diagnosis of personality disorder, and all reported that the diagnosis was borderline personality disorder (BPD), which may now be referred to as emotionally unstable personality disorder. This contrasts with a figure of 1.3% reported in the general population.^29^ This label carries significant stigma, and it is likely that this percentage is an underestimate. Because of a pervasive pattern of instability in affect regulation, impulse control, interpersonal relationships, self-image, and chronic suicidal tendencies,^30^ individuals with BPD can present a challenge to the urological team, and hence a nuanced approach to management is required.

High prevalences of adult trauma (45%) and childhood trauma (35%) were reported in this cohort. Some patients may have elected not to disclose trauma because of the associated stigma. Patients who have experienced sexual, emotional, or physical trauma are more likely to develop psychological comorbidities and unexplained somatic symptoms, and they may also be at a greater risk for developing urinary retention.^31^ Childhood sexual abuse specifically has been shown to be associated with developing affective symptoms such as depression and anxiety, somatization, conversion disorder, and BPD late on in life.^32^ In a recent systematic review of studies that explored LUT dysfunction in survivors of sexual abuse, sexual abuse prevalences ranged between 1.3% and 49.6%.^33^

Individuals with PTSD or complex PTSD are more likely to present with somatization symptoms and medically unexplained symptoms as a result of an adaptive response to psychological distress.^34^ A pelvic examination in a vulnerable individual may trigger a flashback of earlier trauma, and can undermine trust with health care professions and pose a challenge to constructive engagement.

Our study also explored functional neurological disorder, characterized by disabling symptoms that arise from a functional rather than structural disorder. Examples include functional movement disorders, persistent perceptual postural dizziness, functional cognitive disorder, and functional seizures. The prevalence of LUT symptoms in patients with functional neurological symptoms has been reported to be as high as 46% in an online survey of patients,^35^ and OAB symptoms are most commonly reported in patients with functional neurological disorder presenting to a movement disorders service. However, urinary retention has been shown to be particularly prevalent in patients with fixed dystonias, a type of functional neurological symptom disorder.^36^ Functional symptoms generally have been neglected in studies evaluating psychological comorbidities in patients with LUTS. In a retrospective study of women with Fowler’s syndrome, 24% reported functional neurological symptoms.^5^ The current study prospectively enquired about these symptoms and a higher percentage (56%) of women were found to have functional neurological symptoms, and nearly two-thirds (65%) had other functional syndromes such as CFS or IBS. We acknowledge that there may be some bias in the referrals to our center for tertiary referral, which has a focus on EMG and UPP findings.

In the in-depth interview, the respondents were asked if they had either been given a diagnosis of functional neurological disorder or had what had been described as functional neurological symptoms and/or “medically explained symptoms” and/or other functional disorders (with CFS, fibromyalgia, and IBS given as examples of the latter). It is difficult to obtain accurate information about these from a history. Although some patients are clear that they have been given a diagnosis of functional neurological disorder (by a neurologist), some patients said this had been “suggested” by a neurologist or another health care practitioner but to their knowledge was not confirmed. Other patients said the diagnosis of functional neurological disorder had been given but then subsequently “withdrawn,” whilst others said they had been given—but did not accept—the diagnosis of functional neurological disorder. In this study, all of the above were counted as positive for functional neurological disorder. If the patient reported that a diagnosis of a functional syndrome had been suggested by a health care professional then it was counted for this study. As a result, there is likely to be some underreporting. This was an exploratory study, and we recommend that future research attempts to investigate further. We have not reported on treatment for psychological comorbidities because there was considerable uncertainty, for example, whether the patient had mentioned their mental health difficulties to their general practitioner, whether they had been referred or had been advised to self-refer (to an agency), or were currently on a waiting list for assessment or had been assessed but were currently on a waiting list for treatment.

The results of this study highlight that urinary retention is only one symptom amongst several comorbidities that women presenting with CIUR report. Considering the high prevalence of previous trauma, psychological comorbidities, and emotional instability compared to the general population, it is possible that there is a greater vulnerability in this group to developing functional neurological symptoms. Dysfunction of the autonomic nervous system has been reported in functional neurological symptom disorders,^37^ and considering the dual control of the LUT by the involuntary autonomic nervous and voluntary somatic nervous system, it is plausible that LUT functions can also become affected as well, with a loss of functions presenting as urinary retention, perhaps at least in some women, and this needs to be researched further. Furthermore, our current understanding of LUT regulation through the brain-bladder axis suggests a connection between LUT regulation and higher brain centers regulating emotion, arousal, and motivation. For example the amygdala, which plays a critical role in emotional processing, shares several bidirectional connections with regions involved with LUT control including the anterior cingulate cortex, hypothalamus, prefrontal cortex, hippocampus, and the periaqueductal grey. The brain-bladder axis appears to involve an unconscious monitoring of the emotional aspects and safety of voiding behavior.^38^

Amongst females with CIUR, the prevalence of functional neurological symptoms was significantly greater in women with Fowler’s syndrome. They were significantly younger and reported a shorter duration of urological symptoms. This observation also raises the question of whether urinary retention, the hallmark symptom of Fowler’s syndrome, is itself a symptom of functional neurological disorder.

The findings of this study have implications for clinical care. The current approach to managing CIUR is heavily centered around urological management, and our study demonstrates a high prevalence of active psychopathology, including suicidal ideation and personality disorders, and many of these comorbidities can undermine constructive engagement with the urology team. A preliminary study found that whilst screening for psychiatric comorbidities did not predict the outcome of sacral neuromodulation treatment, a psychiatric history was significantly related to the occurrence of adverse events at follow-up.^39^ This finding suggests that further research is needed, to comprehensively document the psychiatric history. A recent International Consultation on Incontinence Research Society paper recommended screening for psychological comorbidities in all age groups.^40^ Sacral neuromodulation has been shown to improve depression,^27^ however this is likely to be addressing affective symptoms that develop after developing urinary retention. Drawing on the current literature around treatments for functional neurological symptoms, PTSD and complex PTSD, and historical cases of urinary retention treated with therapies targeting presumed psychological interventions,^41^ we speculate that a proportion of these patients will benefit from psychotherapy,^42^ although at the present time it would not be possible to predict the impact on LUT functions.

The large sample size is a strength of this study and, as consecutive participants attending the department for tests were approached, the cohort is representative of patients presenting with CIUR to this tertiary referral center. An accurate assessment of psychological comorbidities and functional neurological symptoms was possible through an interview performed by a qualified psychologist. Nevertheless, it is possible that there was some interviewer bias. We acknowledge the lack of a control group is a limitation of this study. As with previous studies, there was a limitation of recall bias.^43^ Many patients who are survivors of trauma, particularly childhood sexual abuse, may find it challenging to recall and share their experiences until they have developed a trusting relationship with the health care provider, and so it is likely that the prevalence of trauma reported in this study is an underestimate.

The important questions, how do psychological comorbidities affect outcomes and is there any evidence that psychological assessment and treatment improves outcomes, are not addressed here. We are collecting follow-up data, including data on treatment outcome, but this is outside the scope of the current paper.

Patients referred to this center for tertiary referral are more complex than those seen in a general urology clinic, and the generalizability of the findings is therefore limited. Notwithstanding these limitations, the findings from this study suggest a substantial burden of psychological comorbidity and functional neurological symptoms amongst women presenting with chronic urinary retention. Future studies should evaluate a possible causal relationship and the impact that bespoke psychological interventions may have in addressing psychological and urological symptoms, and should capture patient-reported outcomes and quality of life using validated instruments.
